# Supplementary material for: Sex differences in anaerobic performance in CrossFit® athletes: a comparison of three different all-out tests
Source: PeerJ. 2025 Feb 5;13:e18930. doi: 10.7717/peerj.18930 (PMC11806908; doi:10.7717/peerj.18930)
Supplement: Supplemental Information 1 [file peerj-13-18930-s001.pdf]

Table S1. Descriptive data of all absolute and relative performance values, gender comparison, effect sizes and percentage of differences

	Group (n=50)			Male (n=25)			Female (n=25)					
	**Mean**	**SD**	**SEM**	**Mean**	**SD**	**SEM**	**Mean**	**SD**	**SEM**	**t**	***d***	**%-dif**
**WG.PP (W)**	700.93	224.08	31.69	895.41	135.75	27.15	506.45	72.75	14.55	0.000	3.57	43.4
**WG.rPP (W/kg)**	9.32	1.68	0.24	10.57	1.16	0.23	8.07	1.09	0.22	0.000	2.22	23.6
**WG.rPP.LM (W/kgLM)**	11.82	1.71	0.24	12.96	1.43	0.29	10.69	1.10	0.22	0.000	1.78	17.5
**WG.rPP.MM (W/kgMM)**	20.09	2.80	0.40	21.79	2.59	0.52	18.39	1.82	0.36	0.000	1.52	15.6
**WG.XP (W)**	511.58	160.89	22.75	653.11	91.80	18.36	370.06	51.92	10.38	0.000	3.80	43.3
**WG.rXP (W/kg)**	6.79	1.13	0.16	7.70	0.63	0.13	5.89	0.71	0.14	0.000	2.71	23.6
**WG.rXP.LM (W/kgLM)**	8.62	1.08	0.15	9.44	0.75	0.15	7.79	0.64	0.13	0.000	2.36	17.5
**WG.rXP.MM (W/kgMM)**	14.66	1.81	0.26	15.88	1.41	0.28	13.43	1.28	0.26	0.000	1.81	15.4
**WG.FI (%)**	54.84	10.31	1.46	56.59	9.24	1.85	53.09	11.20	2.24	0.235	0.34	6.2
**RJT.PP (W)**	977.81	252.19	36.40	1182.21	178.32	36.40	773.42	104.77	21.39	0.000	2.80	34.6
**RJT.rPP (W/kg)**	13.35	1.82	0.26	14.25	1.73	0.35	12.44	1.42	0.29	0.000	1.14	12.7
**RJT.rPP.LM (W/kgLM)**	16.76	1.98	0.29	17.14	2.17	0.44	16.38	1.73	0.35	0.186	0.39	4.4
**RJT.rPP.MM (W/kgMM)**	28.49	3.14	0.45	28.76	3.62	0.74	28.23	2.62	0.53	0.566	0.17	1.8
**RJT.XP (W)**	651.94	233.22	33.66	839.14	150.70	30.76	464.74	123.70	25.25	0.000	2.72	44.6
**RJT.rXP (W/kg)**	8.80	2.16	0.31	10.11	1.49	0.30	7.49	1.95	0.40	0.000	1.51	25.9
**RJT.rXP.LM (W/kgLM)**	10.99	2.40	0.35	12.16	1.88	0.38	9.83	2.32	0.47	0.000	1.11	19.2
**RJT.rXP.MM (W/kgMM)**	18.64	3.79	0.55	20.40	3.14	0.64	16.88	3.61	0.74	0.001	1.04	17.3
**RJT.FI (%)**	59.16	14.33	2.07	53.48	13.04	2.66	64.84	13.49	2.75	0.005	0.86	-17.5
**AST.PP (W)**	1157.41	347.98	49.21	1418.99	295.31	59.06	895.83	132.18	26.44	0.000	2.29	36.9
**AST.rPP (W/kg)**	15.34	2.63	0.37	16.68	2.56	0.51	14.00	1.95	0.39	0.000	1.18	16.1
**AST.rPP.LM (W/kgLM)**	19.71	2.92	0.41	20.49	3.27	0.65	18.93	2.34	0.47	0.059	0.55	7.6
**AST.rPP.MM (W/kgMM)**	33.51	4.88	0.69	34.47	5.82	1.16	32.55	3.58	0.72	0.206	0.40	5.6
**AST.XP (W)**	1016.83	308.88	43.68	1254.09	241.85	48.37	779.57	137.91	27.58	0.000	2.41	37.8
**AST.rXP (W/kg)**	13.45	2.32	0.33	14.74	1.99	0.40	12.16	1.90	0.38	0.000	1.33	17.5
**AST.rXP.LM (W/kgLM)**	17.27	2.48	0.35	18.11	2.53	0.51	16.43	2.16	0.43	0.015	0.71	9.3
**AST.rXP.MM (W/kgMM)**	29.35	4.13	0.58	30.46	4.54	0.91	28.25	3.41	0.68	0.057	0.55	7.3
**AST.FI (%)**	28.17	10.26	1.45	25.51	9.62	1.92	30.82	10.37	2.07	0.067	0.53	-17.2

SD: standard deviation; SEM: standard error of the mean; t: significance of t-student analysis; d: Cohen d effect size; WG: Wingate test; RJT: repeated jump test; AST: anaerobic squat test; PP: peak power in watts; rPP: peak power relative to body mass in watts per kg (W/kg); rPP.LM: peak power relative to lean mass in watts per kg (W/kgLM); rPP.MM: peak power relative to muscle mass in watts per kg (W/kgMM); XP: mean power in watts; rXP: mean power relative to body mass in watts per kg (W/kg); rXP.LM: mean power relative to lean mass in watts per kg (W/kgLM); rXP.MM: mean power relative to muscle mass in watts per kg (W/kgMM); FI: fatigue index.
