# Supplementary material for: Sex differences in anaerobic performance in CrossFit® athletes: a comparison of three different all-out tests
Source: PeerJ. 2025 Feb 5;13:e18930. doi: 10.7717/peerj.18930 (PMC11806908; doi:10.7717/peerj.18930)
Supplement: Supplemental Information 2 [file peerj-13-18930-s002.docx]

STROBE Statement—checklist of items that should be included in reports of observational studies

|  | Item No. | Recommendation | Page  No. | Relevant text from manuscript |
| --- | --- | --- | --- | --- |
| **Title and abstract** | 1 | (*a*) Indicate the study’s design with a commonly used term in the title or the abstract | 1 (30) | A cross-sectional study was conducted |
|  |  | (*b*) Provide in the abstract an informative and balanced summary of what was done and what was found | 1 (31-32)  1 (35) | They were subjected to body composition analysis and three maximal effort tests  Significant differences were found |
| Introduction | | | |  |
| Background/rationale | 2 | Explain the scientific background and rationale for the investigation being reported | 2 (51-52) | differences in athletic performance between men and women have been the subject of study |
| Objectives | 3 | State specific objectives, including any prespecified hypotheses | 3 (106-108) | the present study aims to determine the existing performance differences between sexes in absolute and relative values for body mass, lean body mass and muscle mass in different tests at maximal effort |
| Methods | | | |  |
| Study design | 4 | Present key elements of study design early in the paper | 3 (116-119) | Participants underwent three sessions of performance assessment and one session of BC assessment in the laboratory. In the first session, they underwent BC analysis, and in the subsequent sessions, they underwent maximal exercise testing. |
| Setting | 5 | Describe the setting, locations, and relevant dates, including periods of recruitment, exposure, follow-up, and data collection | 3 (116-117)  3 (119)  4 (122-124) | Participants underwent three sessions of performance assessment and one session of BC assessment in the laboratory  All sessions were separated by at least 48 hours.  They were recruited from an advertisement circulated among CF centers owners in Malaga and surrounding areas |
| Participants | 6 | (*a*) *Cohort study*—Give the eligibility criteria, and the sources and methods of selection of participants. Describe methods of follow-up  *Case-control study*—Give the eligibility criteria, and the sources and methods of case ascertainment and control selection. Give the rationale for the choice of cases and controls  *Cross-sectional study*—Give the eligibility criteria, and the sources and methods of selection of participants | 4 (124-125) | Inclusion criteria were that participants had to train at least three hours per week and had been practicing CF for at least one year |
|  |  | (*b*) *Cohort study*—For matched studies, give matching criteria and number of exposed and unexposed  *Case-control study*—For matched studies, give matching criteria and the number of controls per case |  |  |
| Variables | 7 | Clearly define all outcomes, exposures, predictors, potential confounders, and effect modifiers. Give diagnostic criteria, if applicable | 4 (144-145) | The absolute values of Maximum Power (PP), average (XP) and minimum (MP) were determined for all of them |
| Data sources/ measurement | 8* | For each variable of interest, give sources of data and details of methods of assessment (measurement). Describe comparability of assessment methods if there is more than one group | 4 /136-138)  4 (154)  5 (169-170) | BC analysis was performed by dual-energy X-ray absorptiometry (DXA) (Hologic Inc., Bedford, MA, USA) using Hologic APEX software (version 4.6) and electrical bio-impedance (InBody 770, Cerritos, California, USA).  The original test was performed using a Monark 894E cycle ergometer  Absolute power values for each repetition were extracted from the data provided by the Chronojump® version 2.3 software. |
| Bias | 9 | Describe any efforts to address potential sources of bias | 4 (150-151) | Tests were randomly assigned to avoid sequencing bias. |
| Study size | 10 | Explain how the study size was arrived at | 4 (122-123) | The sample size was established through statistical power analysis |

Continued on next page

| Quantitative variables | 11 | Explain how quantitative variables were handled in the analyses. If applicable, describe which groupings were chosen and why | 5 (177-178) | SPSS version 21.0 software (IBM Corp., Armonk, NY, USA) was used to perform the different analyses  The participants were grouped by sex with the aim of making the comparison. |
| --- | --- | --- | --- | --- |
| Statistical methods | 12 | (*a*) Describe all statistical methods, including those used to control for confounding | 5 (173-175) | The normality of the variables was analyzed using the Shapiro-Wilk test, and the homogeneity of variances using the Levene test. |
|  |  | (*b*) Describe any methods used to examine subgroups and interactions | 5 (176-177) | The Student's t-test was used to compare the mean values of the groups |
|  |  | (*c*) Explain how missing data were addressed |  | missing data were treated as null in the database |
|  |  | (*d*) *Cohort study*—If applicable, explain how loss to follow-up was addressed  *Case-control study*—If applicable, explain how matching of cases and controls was addressed  *Cross-sectional study*—If applicable, describe analytical methods taking account of sampling strategy |  |  |
|  |  | (*e*) Describe any sensitivity analyses |  |  |
| Results | | | | |
| Participants | 13* | (a) Report numbers of individuals at each stage of study—eg numbers potentially eligible, examined for eligibility, confirmed eligible, included in the study, completing follow-up, and analysed |  |  |
|  |  | (b) Give reasons for non-participation at each stage |  |  |
|  |  | (c) Consider use of a flow diagram |  |  |
| Descriptive data | 14* | (a) Give characteristics of study participants (eg demographic, clinical, social) and information on exposures and potential confounders | 5 (173) | Table 1 presents the descriptive data of the sample in mean values |
|  |  | (b) Indicate number of participants with missing data for each variable of interest |  |  |
|  |  | (c) *Cohort study*—Summarise follow-up time (eg, average and total amount) |  |  |
| Outcome data | 15* | *Cohort study*—Report numbers of outcome events or summary measures over time |  |  |
|  |  | *Case-control study—*Report numbers in each exposure category, or summary measures of exposure |  |  |
|  |  | *Cross-sectional study—*Report numbers of outcome events or summary measures | 5 (189-190) | Table S1 shows the numerical values of absolute power, adjusted for body mass, lean mass, muscle mass and fatigue index for each test. |
| Main results | 16 | (*a*) Give unadjusted estimates and, if applicable, confounder-adjusted estimates and their precision (eg, 95% confidence interval). Make clear which confounders were adjusted for and why they were included | 5 (173) | Table 1 presents the descriptive data of the sample in mean values and standard deviations |
|  |  | (*b*) Report category boundaries when continuous variables were categorized |  |  |
|  |  | (*c*) If relevant, consider translating estimates of relative risk into absolute risk for a meaningful time period |  |  |

Continued on next page

| Other analyses | 17 | Report other analyses done—eg analyses of subgroups and interactions, and sensitivity analyses |  |  |
| --- | --- | --- | --- | --- |
| Discussion | | | | |
| Key results | 18 | Summarise key results with reference to study objectives | 6 (238-239) | Regarding the absolute peak powers, the present study's results showed statistically significant differences in all the tests performed (WG, RJT and AST). |
| Limitations | 19 | Discuss limitations of the study, taking into account sources of potential bias or imprecision. Discuss both direction and magnitude of any potential bias | 8 (316-320) | Some limitations can be recognized in the present study |
| Interpretation | 20 | Give a cautious overall interpretation of results considering objectives, limitations, multiplicity of analyses, results from similar studies, and other relevant evidence | 9 (238-329) | However, further research is needed |
| Generalisability | 21 | Discuss the generalisability (external validity) of the study results |  |  |
| Other information | |  | | |
| Funding | 22 | Give the source of funding and the role of the funders for the present study and, if applicable, for the original study on which the present article is based |  | No funding was received to carry out this study. |

*Give information separately for cases and controls in case-control studies and, if applicable, for exposed and unexposed groups in cohort and cross-sectional studies.

**Note:** An Explanation and Elaboration article discusses each checklist item and gives methodological background and published examples of transparent reporting. The STROBE checklist is best used in conjunction with this article (freely available on the Web sites of PLoS Medicine at http://www.plosmedicine.org/, Annals of Internal Medicine at http://www.annals.org/, and Epidemiology at http://www.epidem.com/). Information on the STROBE Initiative is available at www.strobe-statement.org.
